# Renal artery stenosis due to neurofibromatosis type 1: case report and literature review

**DOI:** 10.1186/2047-783X-19-17

**Published:** 2014-03-28

**Authors:** Lian Duan, Kai Feng, Anli Tong, Zhiyong Liang

**Affiliations:** 1 Key Laboratory of Endocrinology, Ministry of Health; Department of Endocrinology, Peking Union Medical College Hospital, Peking Union Medical College and Chinese Academy of Medical Sciences, Beijing 100730, China; 2 Department of Pathology, Peking Union Medical College Hospital, Peking Union Medical College and Chinese Academy of Medical Sciences, Beijing 100730, China

**Keywords:** Neurofibromatosis type 1, hypertension, renal artery stenosis

## Abstract

**Background:**

Neurofibromatosis type 1 (NF1) is a relatively common autosomal dominant disorder. The most common vascular abnormality in patients with NF1 is bilateral or unilateral renal artery stenosis.

**Case report:**

A 16-year-old boy presented with a headache of 4-year duration and was found to be moderately hypertensive. On physical examination, axillary freckling and multiple café-au-lait spots were revealed over the trunk, while numerous small nodules were palpable on the limbs. Biopsy of subcutaneous nodule showed neurofibroma. Lisch nodules were identified on slit-lamp examination and grade I hypertensive retinopathy was present on fundoscopy. Clinical laboratory investigations revealed that renal and liver function tests, blood cells count, urinalysis, serum electrolytes, serum levels of renin and aldosterone, and 24-hour urine levels of catecholamines were all within normal ranges. Abdominal ultrasound and CT were normal. Both kidneys were of normal size. CT angiography showed right renal artery stenosis (>90%) at the ostium. The final diagnosis of NF1 with right renal artery stenosis and secondary hypertension was then made. The patient was treated with Procardin (30 mg/d) and improved with a significant decline in blood pressure. The main outcomes were to control blood pressure without necessarily proceeding with PTRA. We also present a review of the literature.

**Conclusions:**

NF1 may present with hypertension due to renal artery stenosis in children. All young patients (<30 year) with hypertension should be clinically screened for secondary causes of hypertension, including NF1, so that renal revascularization can be offered before permanent end organ damage has occurred. First-line management using medication alone could be appropriate, keeping the interventional options for when the patient's condition deteriorates.

## Background

Neurofibromatosis type 1 (NF1), formerly known as von Recklinghausen’s disease, is an autosomal dominant disorder with a birth incidence of 1 in 2,500 to 3,000, independently of ethnicity and gender [1]. The gene responsible for NF1 is located on chromosome 17q11.2, and its protein product, neurofibromin, is ubiquitously expressed at high levels in the nervous system, and functions as a tumor suppressor.

Diagnosis is usually made on the basis of: 1) café-au-lait macules; 2) neurofibromas; 3) Lisch nodules of the iris; 4) axillary freckling; 5) optic pathway gliomas; 6) distinctive bone lesions; and 7) a first-degree relative with NF1 [2]. Other clinical manifestations are abnormalities of the cardiovascular, gastrointestinal, renal and endocrine systems, cognitive deficit, and malignancies of the peripheral nerve sheath and central nervous system [2].

The cardiovascular features of NF1 may include congenital heart disease, vasculopathy and hypertension. Vasculopathies are the second cause of death in NF1 patients [3]. The incidence of hypertension (>95th percentile for age and gender) in patients with NF1 is approximately 16% [4], and is mostly due to renal artery stenosis in children [5,6], followed by coarctation of the aorta and pheochromocytomas [2,7,8]. An annual blood pressure screening and heart examination are therefore warranted [2]. Any abnormal finding should be investigated with renal arteriography and 24-h urinary excretion of catecholamines and metabolites [9]. Magnetic resonance imaging and computed tomography (CT) should be performed only after biochemical findings are observed [2,9].

The current management of NF1 focuses on symptomatic treatment. Therefore, early diagnosis of renal artery stenosis is important, because curative treatment can prevent the adverse consequences of hypertension. The blood pressure target in NF1 patients should be <140/90 mmHg [9,10]. Renal angioplasty may be necessary, but may be insufficient to resolve hypertension, and drugs may be necessary [11-13].

We present and discuss the case of a 16-year old boy diagnosed with NF1 and unilateral renal artery stenosis. We focused on the first-line medical management, and reviewed the literature.

## Case presentation

**Figure 1:**
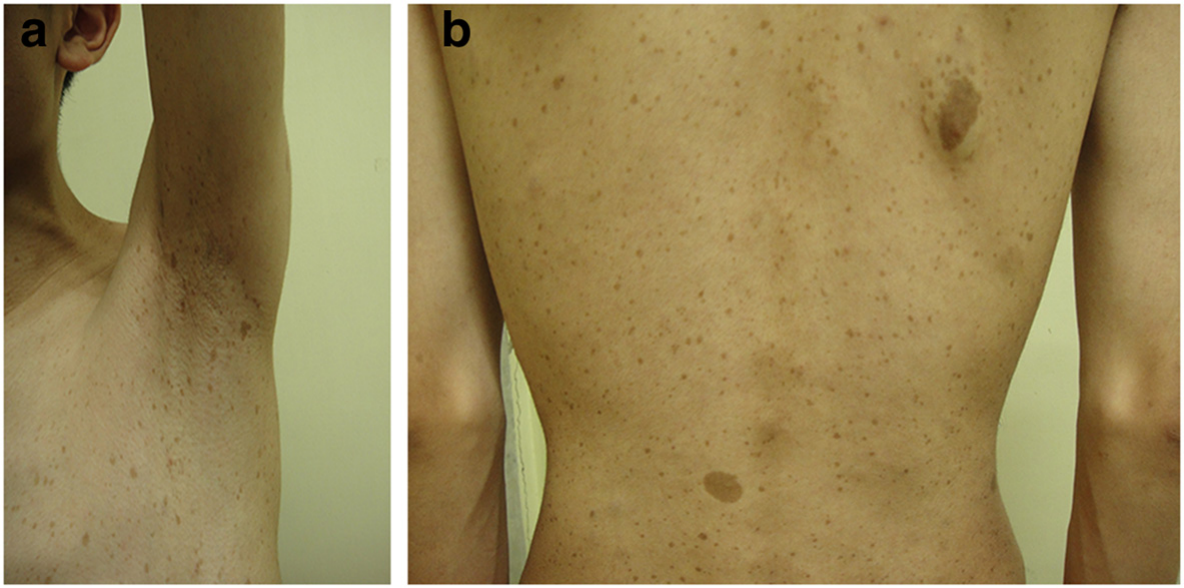
**The special signs of the patient.** Axillary freckling **(a)** and multiple caféau-lait spots** (b)** spread over the skin of the trunk.

A 16-year-old boy was admitted to the Peking Union Medical College Hospital in June 2011, and presented with an intermittent headache of 4-year duration and was found to be moderately hypertensive. It was his first lifetime visit to a doctor. On physical examination, axillary freckling and multiple café-au-lait spots ≥5 mm in size were revealed, spread over the skin of the trunk (Figure 1), while numerous small nodules ≤1 cm were palpable in the limbs. The pigmentation was present when he was born. He was always lean because of low appetite, and had always achieved poor academic performance. His height was 166 cm (10th to 25th percentile for same age and gender [14]), weight was 43 kg and body mass index (BMI) was 15.6 kg/m^2^.

**Figure 2:**
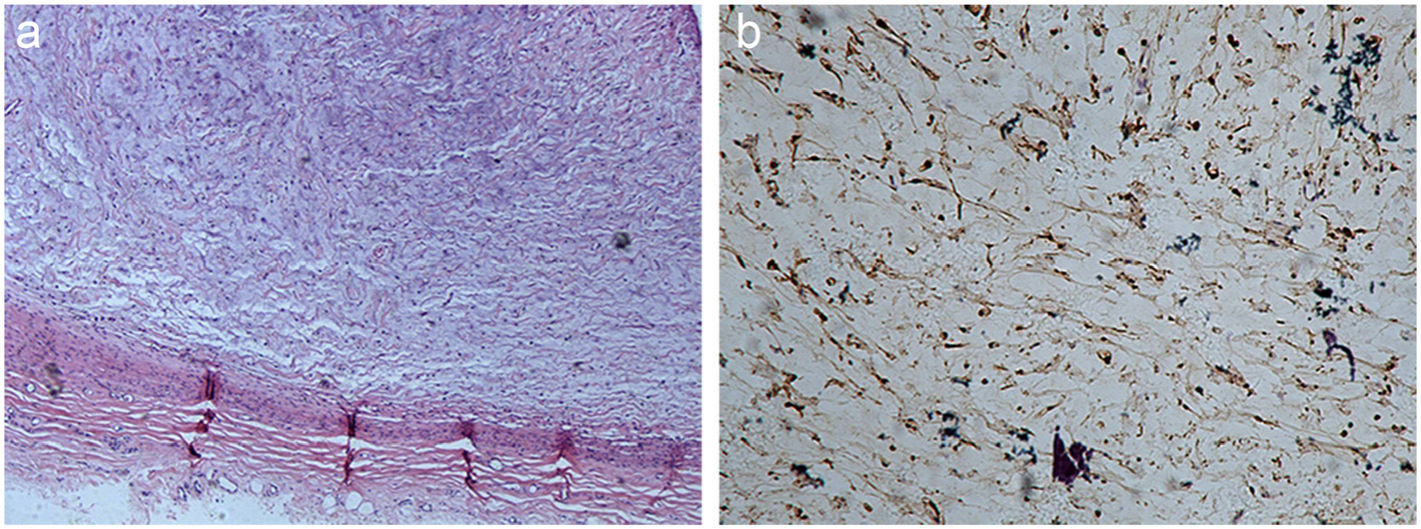
**Pathology result of subcutaneous nodules. (a)** Hematoxylin and eosin (HE) staining of subcutaneous nodules revealed the diagnosis of neurofibroma accompanied by mucinous degeneration (magnification × 40). **(b)** Immunohistochemistry staining for S-100 protein was positive (magnification × 40).

Interestingly, his first-degree family history revealed no case of hypertension, cardiovascular diseases or NF1. Biopsy of a subcutaneous nodule resulted in diagnosis of a neurofibroma, which was confirmed by S100 protein immunoreactivity of many tumor cells (Figure 2). Lisch nodules were identified on slit-lamp examination and grade I hypertensive retinopathy was present on fundoscopy. All peripheral pulses were palpable and blood pressure was recorded in all four limbs using a mercury sphygmomanometer (three times, and using the mean): right arm = 150/100 mmHg, left arm = 150/95 mmHg, right leg = 175/120 mmHg and left leg = 170/115 mmHg. According to the 2010 blood pressure reference standard in Chinese children and adolescents, the 95th and 99th percentile of SBP (Systolic Blood Pressure) for 16-year old boys are 130 mmHg and 141 mmHg, and the 95th and 99th percentile of DBP (Diastolic Blood Pressure) are 85 mmHg and 91 mmHg [15].

Clinical laboratory investigations revealed that renal and liver function tests, blood cell counts, urinalysis, serum electrolytes, serum levels of renin and aldosterone, and 24-h urine levels of catecholamines were all within normal ranges. Peripheral blood renin level was 0.34 ng/ml/h measured in the supine position, and 1.56 ng/ml/h measured in the standing position.

**Figure 3:**
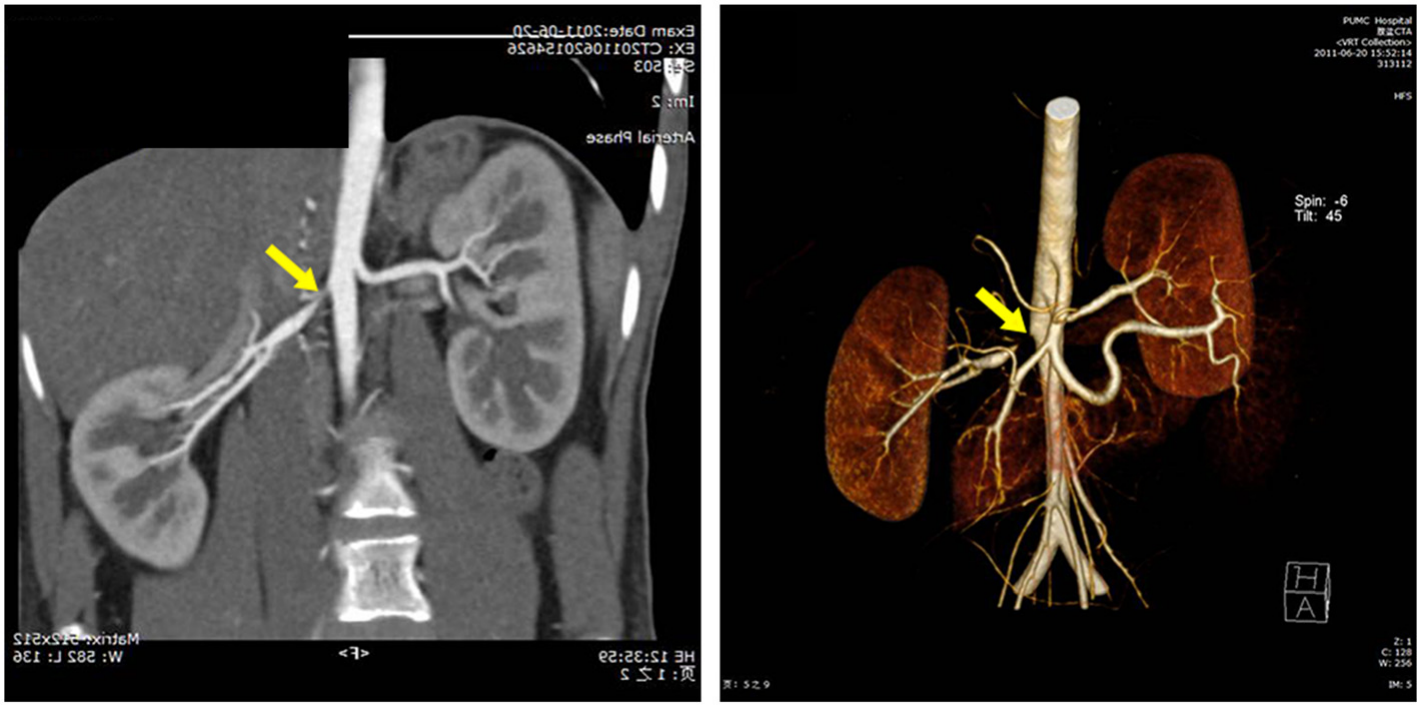
**Computed tomography angiography of the renal vessels.** The initial segment of the right artery was almost occluded, but there was a collateral circulation from the subphrenic artery. Blood flow was 36.7 ml/minute in the right renal, and 57.1 ml/minute in the left.

Ultrasound examination of the abdomen was normal. Both kidneys were of normal size (the right kidney was 10.1 × 4.0 × 4.1 cm and the left kidney was 11.3 × 3.9 × 3.8 cm; cortical thickness was 0.6 cm for the right kidney, and 0.5 cm for the left). Abdominal CT did not show any adrenal or abdominal mass. CT angiography showed right renal artery stenosis (>90%) at the ostium, but also that there was collateral circulation from the subphrenic artery (Figure 3). Blood flow was 36.7 ml/minute in the right renal, and 57.1 ml/minute in the left. The final diagnosis of NF1 with right renal artery stenosis and secondary hypertension was then made.

The patient was treated with oral nifedipine sustained-release preparation (30 mg once a day), and improved with a significant decline in blood pressure (120 to 140/80 to 90 mmHg) after one week. Because: 1) the patient was young; 2) the right renal size was approximately normal; 3) the mild decrease in right renal blood flow was attributable to the collateral circulation; 4) the normal renin levels; and 5) improvements in blood pressure using drugs, the patient and his parents refused percutaneous transluminal renal angioplasty (PTRA) at this time. Therefore, the patient was discharged from our hospital after 1 month. The nifedipine treatment was continued and the patient was simply followed up with close monitoring of blood pressure, and renal function at least once every three months in our outpatient department. PTRA was kept as an option should the patient's condition deteriorate. Blood pressure was well-controlled during one year follow-up. The patient was lost to follow up after one year.

## Discussion

We reported the case of a 16-year-old boy who presented with hypertension. He was diagnosed with NF1 and unilateral renal artery stenosis. Medical treatment alone improved his condition, and interventional treatment was kept as an option for an eventual deterioration of his condition. Therefore, medical treatment should be the initial approach to control hypertension in order to allow maximal body growth before any intervention. However, children with sustained hypertension despite optimal medical management should undergo intervention to avoid the progression of end-organ damage. Consequently, we monitored his blood pressure, renal function and renal blood flow.

Renovascular hypertension is an important cause of secondary hypertension in children, and is responsible for 3.0% to 8.5% of pediatric hypertension [16,17]. The most common causes of renovascular hypertension in children reported in the western literature are fibromuscular dysplasia (FMD) and midaortic syndrome [16]. However, Takayasu’s arteritis is the most important cause of hypertension in Asian children [18]. Renal artery stenosis may also occur in association with NF1, Williams' syndrome, Marfan's syndrome, congenital Rubella syndrome, Kawasaki disease, and Crohn’s disease [19].

NF1 typically presents with café-au-lait spots, multiple neurofibroma, axillary freckling, and ocular Lisch nodules [2]. About 16% of patients with NF1 develop hypertension [4], either essential hypertension or secondary hypertension due to renovascular disease, coarctation of the abdominal aorta or pheochromocytoma [2,5-8]. In children and young adults with NF1, the most frequent cause of hypertension is renovascular disease, which occurs seven times more frequently than pheochromocytoma [9]. Renal artery stenosis in NF1 is usually ostial in location, as was seen in our case [9]. In contrast with fibromuscular dysplasia where 95% of all stenoses are found in the distal two-thirds of the renal artery, more than 50% of all stenoses in NF1 are located at the ostia [9].

Greene *et al*. originally described two basic categories of stenosis in NF1. The first involves larger vessels such as the aorta, carotid and proximal renal arteries, which are surrounded by neurofibromatosis or ganglioneuromatous tissue. Intimal proliferation, thinning of the media and fragmentation of elastic tissue may lead to stenosis or aneurysm formation. The second type is unrelated to neural malformation, but probably reflects dysplasia of small vessels. Lesions occur in many arteries and involve the minute intrarenal branches. Using electron microscopy, Greene *et al*. identified smooth-muscle elements within the abnormal cells and concluded that the vascular lesion is a form of mesodermal dysplasia [20]. The case presented here probably belongs to the first category.

Renovascular hypertension results in decreased blood flow to the kidney, resulting in increased production of renin. The aim of angioplasty is therefore to restore blood flow to the kidney, thereby decreasing renin production [21]. Treatment modalities in renovascular hypertension secondary to NF1 involve a combination of drug therapy, PTRA, and surgery [9-13]. Indeed, Kimura *et al*. reported good outcomes using renal artery repair in children, including NF1 patients [12,13]. Malav *et al*. [6] reported good outcomes using antihypertensive drugs and renal angioplasty, whereas Ueda *et al*. [11] and Booth *et al*. [22] reported persistent high blood pressure after renal angioplasty. Oderich *et al*. [3] reported safe, effective and durable outcomes of surgical management of NF1-associated hypertension. However, it is our opinion that medical treatment should be the initial approach for controlling hypertension to allow maximal body growth before any surgical intervention, which is also advocated by Tullus *et al*. [21]. However, we agree that poorly controlled hypertension using medical therapy is undesirable in children because of the progression of end-organ damage. A review of 16 NF1 patients with renal artery stenosis who underwent PTRA had a 33% success rate with PTRA for primary stenosis. The success rate was higher (67%) in post-surgery residual stenosis [22]. Because of the absence of major complications and no adverse effect on subsequent vascular reconstruction, PTRA should be considered as first-line treatment for renal artery stenosis in NF1 pediatric patients only when clinically indicated. In both NF1 and Takayasu’s arteritis, the presence of ostial stenosis and long-segment disease predisposes to restenosis. Surgical treatment of renal artery stenosis by reimplanting the renal artery on the aorta, or bypassing the obstruction using the saphenous vein or splenic artery, have been reported with good results [17]. Although restenosis after PTRA is not uncommon, there are several advantages of PTRA over surgery. Reduced morbidity from the lack of a surgical incision and reduced hospital stay are desirable at all ages. Furthermore, PTRA does not interfere with subsequent surgery if it is needed. Stent angioplasty is technically successful and can improve hypertension in selected patients. However, arterial stenting is generally avoided in the pediatric population unless angioplasty results in a flow-limited dissection, complete recoil of stenosis, or focal arterial rupture [23].

Han and Criado [24] reviewed 13 papers including 49 patients with NF1 and renovascular hypertension, and who were treated with medication, surgery, or percutaneous transluminal angioplasty (PTA) or coil embolization. All patients received medical therapy. Eight patients were treated with medication alone, 10 had a nephrectomy, 13 had surgical revascularization procedures, 16 had a PTA, and 1 patient had transluminal embolization of an aneurysm. Medication alone improved the condition of 56% of the patients, but also led to failure in 22%. However, this rate was similar to the failure rates of surgery (22%) and PTRA (35%).

In addition to renal artery stenosis, vascular lesions in NF1 may involve mesenteric vessels, aorta, and cerebral vessels. NF1 vasculopathy tends to be progressive and is one of the leading causes of death in this population [3]. Therefore, careful long-term follow up is therefore required.

## Conclusions

NF1 may present with hypertension due to renal artery stenosis in children. A wider appreciation of this entity is warranted for early diagnosis and appropriate, prompt treatment. In summary, NF1 is a disorder usually associated with bilateral or unilateral renal artery stenosis. All young patients (<30 years) with hypertension should be clinically screened very early for secondary causes of hypertension, including NF1, so that renal revascularization can be offered before permanent end-organ damage has occurred.

## Consent

Written informed consent was obtained from the patient for publication of this Case report and any accompanying images. A copy of the written consent is available for review by the Editor-in-Chief of this journal.

## Abbreviations

CT: Computed tomography; FMD: Fibromuscular dysplasia; NF1: Neurofibromatosis type 1; PTRA: Percutaneous transluminal renal angioplasty.

## Competing interests

The authors declare that they have no competing interests.

## Authors’ contributions

All authors participated in the preparation of the manuscript. LD collected all clinical data. All authors read and approved the final manuscript.
